# Glassy Carbon Electrocorticography Electrodes on Ultra-Thin and Finger-Like Polyimide Substrate: Performance Evaluation Based on Different Electrode Diameters

**DOI:** 10.3390/ma11122486

**Published:** 2018-12-07

**Authors:** Maria Vomero, Elena Zucchini, Emanuela Delfino, Calogero Gueli, Norma Carolina Mondragon, Stefano Carli, Luciano Fadiga, Thomas Stieglitz

**Affiliations:** 1 Institute of Microsystem Technology (IMTEK), Laboratory for Biomedical Microtechnology, Georges-Koehler-Allee 102, D-79110 Freiburg, Germany; guelic@tf.uni-freiburg.de (C.G.); carolina.mondragon@student.uni-freiburg.de (N.C.M.); thomas.stieglitz@imtek.uni-freiburg.de (T.S.); 2 Cluster of Excellence BrainLinks-BrainTools, University of Freiburg, Georges-Koehler-Allee 80, 79110 Freiburg, Germany; 3 Center for Translational Neurophysiology of Speech and Communication, Istituto Italiano di Tecnologia (IIT), Via Fossato di Mortara 17/19, 44121 Ferrara, Italy; Elena.Zucchini@iit.it (E.Z.); Emanuela.Delfino@iit.it (E.D.); Stefano.Carli@iit.it (S.C.); Luciano.Fadiga@iit.it (L.F.); 4 Section of Human Physiology University of Ferrara, Via Fossato di Mortara 17/19, 44121 Ferrara, Italy; 5 Bernstein Center Freiburg, University of Freiburg, Hansastrasse 9a, 79104 Freiburg, Germany

**Keywords:** glassy carbon, pyrolysis, neural implants, ECoG, conformability, polyimide, poly(3,4-ethylenedioxythiophene) (PEDOT)

## Abstract

Glassy carbon (GC) has high potential to serve as a biomaterial in neural applications because it is biocompatible, electrochemically inert and can be incorporated in polyimide-based implantable devices. Miniaturization and applicability of GC is, however, thought to be partially limited by its electrical conductivity. For this study, ultra-conformable polyimide-based electrocorticography (ECoG) devices with different-diameter GC electrodes were fabricated and tested in vitro and in rat models. For achieving conformability to the rat brain, polyimide was patterned in a finger-like shape and its thickness was set to 8 µm. To investigate different electrode sizes, each ECoG device was assigned electrodes with diameters of 50, 100, 200 and 300 µm. They were electrochemically characterized and subjected to 10 million biphasic pulses—for achieving a steady-state—and to X-ray photoelectron spectroscopy, for examining their elemental composition. The electrodes were then implanted epidurally to evaluate the ability of each diameter to detect neural activity. Results show that their performance at low frequencies (up to 300 Hz) depends on the distance from the signal source rather than on the electrode diameter, while at high frequencies (above 200 Hz) small electrodes have higher background noises than large ones, unless they are coated with poly(3,4-ethylenedioxythiophene)-poly(styrenesulfonate) (PEDOT:PSS).

## 1. Introduction

**Figure 1:**
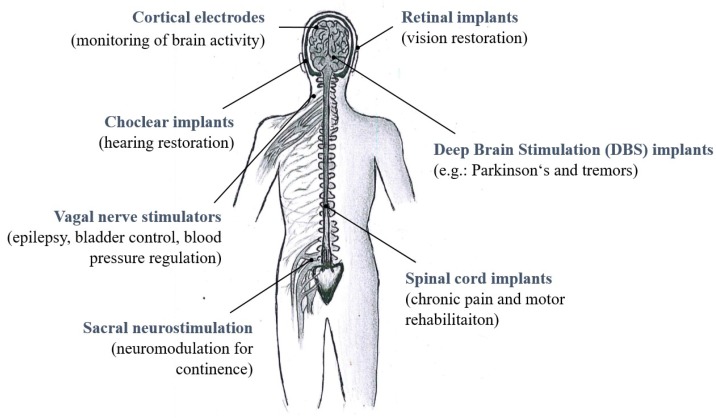
Sketch with examples of different types of neural implants in different sections of the human nervous system with examples of main clinical applications.

Neural implants are assistive devices used to record and stimulate specific regions of the nervous system to help restore functions lost as a result of a neural damage [1,2]. Depending on the type of damage and on the targeted anatomical area, design and location of the implants changes (Figure 1) and can be tailored for reaching the best compromise between invasiveness and function restoration. Although they are considered minimally invasive when compared to penetrating devices, microelectrode arrays (MEAs) for ECoG still elicit foreign-body response and glial scar formation that can—in the long term—isolate them from the nerve cells [3,4,5]. One way to minimize their encapsulation is to reduce the substrate footprint on the brain surface and promote the diffusion of soluble factors through the devices by optimizing their design [6,7]. Another important aspect is the conformability of the ECoG MEAs to the brain surface, to allow adaptation to its curvature and allow the electrodes to closely connect to the living tissue and potentially enable the recordings of low- and high-frequency signals [8,9]. Electrode performance, in fact, does not only depend on their surface chemistry and morphology but also on the mechanical tissue–electrode interaction and on the quality of the interface they form. When the device’s substrate material (e.g., silicone rubber or polyimide) is too thick and does not naturally conform to the curvilinear and soft brain tissue, the low electrical quality of the biotic/abiotic interface can potentially compromise the quality of the recordings and can eventually result in tissue loss [9,10].

On the other hand, for improving the electrical performance of neural prosthesis, the electrode material choice remains critical and should not be overlooked. GC, for instance, has lately been introduced in the field of neural engineering thanks to its incorporation into polyimide-based devices [11,12,13] and to its electrochemical stability, biocompatibility and great short- and long-term reliability [14,15,16,17,18]. Such a form of carbon—which can be obtained by pyrolysis of SU-8 or other phenol-formaldehyde resins—is currently one of the most interesting electrode materials because of its capability to simultaneously serve as recording, stimulation and biosensing platform [19]. 

The undeniable potential of carbon as an electrode material is thought to be restrained by the limitation in the miniaturization of the electrodes themselves, due to the material’s relatively low conductivity and high dimensional shrinkage during pyrolysis [20]. By increasing the surface and the structural biocompatibility of the device and focusing on the mechanical and chemical interaction of the whole implant with the host tissue—rather than modifying the surface chemistry and morphology of the electrodes to lower the impedance—it is, however, possible to improve the quality of the interface and of the acquired signal by reducing the gap between the electrode and the nerve cells. Therefore, in this study, GC electrodes with different diameters (50, 100, 200 and 300 μm) are incorporated into a highly conformable 8-μm-thick polyimide substrate with a fenestrated and finger-like footprint to evaluate their in vitro and in vivo performances, with and without PEDOT:PSS coating. In addition, the shrinkage of the electrode structures after pyrolysis is taken into account, and particular attention is given to how the disk-shaped SU-8, the precursor used to obtain GC microelectrodes, deforms after the carbonization bake (i.e., pyrolysis). Although pyrolysis-related shrinkage depends on many factors—like the final temperature reached during the process [21,22], the presence of nitrogen or vacuum in the chamber [23] and the aspect ratio of the structures [20]—for certain geometries it seems to be highly predictable and reproducible [20,24]. During the pyrolysis of disk-shape structures with low aspect ratios, in fact, degassing mainly occurs through their top surface (rather than through the lateral surface) and thus the vertical shrinkage (or height shrinkage) is expected to be greater than the shrinkage in diameter. When the aspect ratio increases, later shrinkage also increases and vertical shrinkage tends to decrease [20]. A change in the shape of the disks is also expected when they adhere to a substrate during the carbonization process (i.e., SU-8 disks on a silicon wafer): they sag in the middle and take a ‘volcano’ shape due to degassing and residual stresses during pyrolysis. It remains unclear, however, if these aspects have an effect on the GC electrode’s electrochemical behavior and recording performance. In this manuscript, we investigate and correlate GC microelectrodes’ dimensions to their ability to record neural activity in low- and high-frequency domains, with the aim of understanding how the design of the precursor polymer and its pyrolysis-dependent shrinkage affect the performance of the ECoG arrays in vitro and in vivo. The evaluation of GC microelectrodes is performed in the best-case scenario of highly conformable polyimide substrate and thus of mechanically stable biotic/abiotic interface.

## 2. Materials and Methods 

### 2.1. Fabrication of the ECoG Arrays and PEDOT:PSS Deposition

**Figure 2:**
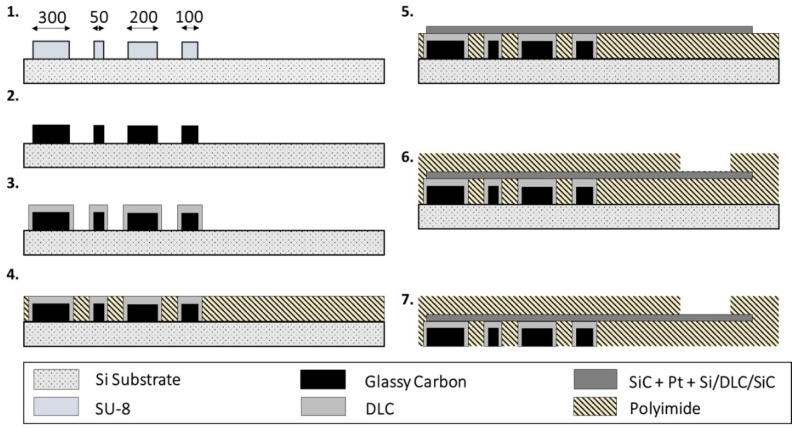
Microfabrication process used to fabricate the ultra-conformable polyimide-based ECoG devices with GC electrodes: (**1**) Lithographically patterned SU-8 disks with diameters of 50, 100, 200 and 300 µm and height of 13 µm. (**2**) Pyrolysis of SU-8 structures (1000 °C) to obtain GC electrodes. (**3**) PECVD (plasma-enhanced chemical vapor deposition) of 50 nm DLC (diamond-like carbon) onto GC electrodes. (**4**) First layer of polyimide (PI, 4 µm), patterned to open the vias to the GC electrodes. (**5**) PECVD of 50 nm SiC (silicon carbide) on the tracks of the devices, followed by evaporated platinum (Pt, 300 nm) and 50 nm Si/DLC/SiC. (**6**) Second layer of PI (4 µm), patterned to access the metal bump pads. (**7**) Buffered oxide etch (BOE) for releasing the devices from the silicon wafer.

The ECoG devices used for this study were fabricated using a microfabrication protocol described elsewhere in detail with all process parameters [15] and sketched in Figure 2. 

In short, SU-8 3010 (MicroChem, Westborough, MA, USA) was spun on a silicon wafer and photolithographically patterned in 4 × 4 arrays of disk-shaped structures with diameters of 50, 100, 200 and 300 µm and height of 13 µm. They were then pyrolyzed in a furnace (PEO 601, ATV-Technologie GmbH, Vaterstetten, Germany) at 1000 °C in N_2_ atmosphere and coated with 50 nm of methane-based PECVD DLC. Polyimide (U-Varnish, UBE, Tokyo, Japan) was spin coated onto the substrate and cured at 450 °C to a thickness of 4 µm and a second photolithography step was implemented to define openings on the backside of the carbon electrodes. After, a third photolithography step (AZ5214E Microchemicals GmbH, Ulm, Germany) was undertaken to define the tracks for the electrical routing and a 50 nm layer of SiC was deposited, followed by 300 nm of platinum and 50 nm of Si, DLC and SiC (PECVD deposited). A second layer of polyimide was spun onto the tracks (4 µm) for insulation and patterned to access the interconnection sites. Finally, the devices were released from the silicon wafer through a buffered oxide etching process. The electrodes of 2 ECoG devices were then coated with PEDOT:PSS. PEDOT:PSS was prepared by anodic electrodeposition from a solution of 3,4-ethylenedioxythiophene (EDOT, 0.02 M) and poly(sodium 4-styrene sulfonate) (NaPSS, 0.1 M) in water. The electrochemical deposition was carried out in potentiodynamic mode, in the potential range of 0–0.95 V, with a scan rate of 100 mV/s, for a total of 20 cycles per electrode. A Reference 600 potentiostat (Gamry Instruments, Warminster, PA, USA) connected to a three-electrode electrochemical cell was used for the electrodeposition; a Pt wire and a Ag/AgCl (+0.197 V vs. Normal hydrogen electrode—NHE) were set as counter and reference electrode, respectively.

### 2.2. Shrinkage Measurements

The thickness of disk-shaped carbon structures, before and after pyrolysis, was determined using a mechanical profilometer (P-11 Tencor, Milpitas, CA, USA). For this test, SU-8 3010 was spun on a 4-inch silicon wafer and patterned in the shape of disks with diameters of 40, 100, 220 and 340 µm and height of 13 µm, leaving exposed the silicon among them. A surface profile of the structures (n = 5 per type) was taken by moving the profiler stylus across the structures. Measurements were repeated in the middle and in 4 other spots at the edge of the wafers, and then averaged per each diameter. Shrinkage was calculated in percentage terms using Equation (1) and the ratio between geometrical lateral area and surface area (L/S Area Ratio) before pyrolysis was used to categorize the structures as ‘short’ (when < 1) or ‘intermediate’ (when > 1). Before pyrolysis, the dimensions of the structures are the ones determined by design (and therefore are not expected to differ from one sample to another).

(1) $$S=\;\frac{height\;before\;pyrolysis-height\;after\;pyrolysis}{height\;before\;pyrolysis}\times 100$$

### 2.3. Electrochemical Characterization 

Carbon electrodes were electrochemically characterized by impedance spectroscopy (EIS) and cyclic voltammetry (CV) measurements in 0.01 M phosphate buffered saline—PBS (Sigma-Aldrich, Saint Louis, MO, USA). For the EIS test, a sinewave with an amplitude of 10 mV was superimposed onto the open circuit potential while varying the frequency from 1 to 10^5^ Hz. For the CV measurements, instead, the working electrode potential was swept between 1.1 and −0.9 V versus Ag/AgCl while maintaining a scan rate of 100 mV s^−1^. EIS and CV were performed using a potentiostat/galvanostat (Solartron Analytical, Ametek Scientific Instruments, Leicester, UK) connected to a three-electrode electrochemical cell with a platinum counter electrode and an Ag/AgCl reference electrode.

### 2.4. Biphasic Pulse Stimulation 

The different-diameter GC electrodes were subjected to 10 million cathodic-first biphasic pulses in PBS, maintaining a charge density of about 0.15 mC/cm^2^: this resulted in current amplitudes of 500, 220, 56 and 14 µA for the 300, 200, 100 and 50 µm diameter electrodes, respectively, and a 200 µs cathodic half-phase period. Similar stimulation protocols were previously used to investigate the ability of GC electrodes to withstand electrical stimulation [15,16]. It was, in fact, found that after an initial functionalization of the GC surface (first 8 million pulses), no further change in impedance should be expected for at least 15 million biphasic pulses [16] and thus such a protocol can be used for achieving an electrochemical steady-state of the GC electrodes. 

### 2.5. X-Ray Photoelectron Spectroscopy (XPS)

XPS measurements were performed using a K-Alpha+ XPS spectrometer (Thermo Fisher Scientific, East Grinstead, UK). For the data acquisition and processing, Thermo Avantage software (Thermo Fisher Scientific, Waltham, MA, USA) was used [25]. All samples were analyzed using a microfocused, monochromated Al Kα X-Ray source (30–400 µm spot size). The spectra were fitted with one or more Voigt profiles (binding energy uncertainty: +/− 0.2 eV). The analyzer transmission function, Scofield sensitivity factors [26] and effective attenuation lengths (EALs) for photoelectrons were applied for quantification. EALs were calculated using the standard TPP-2M formalism [27]. All spectra were referenced to the C1s peak of hydrocarbon at 285.0 eV binding energy. Images of the carbon electrodes before and after stimulation were taken using a Leica microscope (Leica Microsystems, Wetzlar, Germany).

### 2.6. Animals and Surgical Procedure 

Five adult Long Evans rats (400–500 g) were used for acute in vivo experiments. Aiming to record somatosensory evoked potentials, animal surgery and implantation of the ECoG electrodes over the rat barrel cortex (A-P: between −1 mm and −4 mm from bregma, M-L: between −3 mm and −5 mm from the midline) were carried out following a procedure previously described [14]. The experimental plan was designed in compliance with the guidelines established by the European Communities Council (Directive 2010/63/EU, Italian Legislative Decree n. 26, 4/3/2014) and the protocol was approved by the Ethics Committee for animal research of the University of Ferrara and by the Italian Ministry of Health (authorization n 332/2015-PR). 

### 2.7. Somatosensory Evoked Potentials Recording 

Neural signal recordings were performed using a Tucker Davis Technologies (TDT) multi-channel recording system 3 (Tucker Davis Technologies, Alachua, FL, USA) including: the ZIF-Clip® headstage with unity (1×) gain, the RZ2 real-time processor and the PZ2-256 battery-powered preamplifier. In order to reduce electromagnetic noise, the experimental setup was placed in a Faraday cage. Data were digitized at a sample rate of 12,207 samples/s at 18-bit resolution and transferred from RZ2 processor to computer by fast fiber optic connection. As required by the single-ended headstage configuration [28], reference and ground pins of the headstage were tied together and connected to a skull screw. For each rat, several positions and orientations of the ECoG devices over the barrel cortex were investigated in order to evaluate the device reliability in detecting a well-known neurophysiological signal as the somatosensory evoked potentials (SEPs). To mechanically elicit the SEPs, a vibrating system was used to produce a multi-whiskers deflection along the horizontal plane. The whiskers contralateral to the craniotomy were shortened and inserted in a velcro strip attached to a rod moved by a shaker (Type 4810 mini shaker; Bruel & Kjaer, Naerum, Denmark) controlled by a National Instruments board (Austin, TX, USA). When dealing with single whisker stimulation, in place of the velcro strip, the whisker of interest was inserted into a needle attached to the rod. In both cases, the deflection stimulus, consisting of a sine waveform of 12 ms duration, was delivered at 10 Hz. The stimulation amplitude was coincident with a multi-whisker deflection of 500 µm. Each deflection stimulus was repeated 100 times and separated from the others by a 4 s pause.

### 2.8. In Vivo Data Analysis 

In this study, signal processing was carried out using built-in tools and custom-made functions, developed in a MATLAB environment (version 9.3, Mathworks, Natick, MA, USA). Raw data were segmented into trials of 80 ms time-locked to the stimulation (ranging from 20 ms before the onset to 60 ms after it). Furthermore, a time interval 100 ms long before the stimulation was considered for each trial as background noise to compute the signal-to-noise ratio. Time series were bandpass-filtered to 200–1000 Hz in order to extract the high-frequency components of the SEP (HFCs), using a digital zero-phase 4th-order Butterworth. Then, the filtered trials were averaged for the visualization and the analysis of the SEPs in the different experimental conditions.

#### 2.8.1. Estimation of HFCs’ Spatial Distribution

In order to better understand the spatial distribution of the evoked potentials, the analytic upper envelope of the averaged HFCs was considered. The peak amplitude of the envelope was used as a feature to quantify the size of the evoked responses recorded from each electrode. To model the spatial distribution of the SEPs across the array, the features were spatially interpolated in a bidimensional map, by fitting the 4 × 4 peak amplitude matrix with a thin-plate spline model available in the MATLAB (Mathworks, Natick, MA, USA) Curve Fitting Toolbox.

#### 2.8.2. Signal-to-Noise Ratio

To assess the quality of the recordings for the HFCs of each electrode, we calculated the ratio:

(2) $$SNR_{el}=median\left(\frac{S_{i,\;el}}{2*std\left(N_{i,\;el}\right)}\right),$$

where *S_i,el_* is the peak-to-peak amplitude of the i^th^ trial for the electrode *el*, and *N_i,el_* is the standard deviation of 100 ms before the i^th^ stimulation for the same electrode. Then, *SNR_el_* is computed as the median value over trials.

## 3. Results

### 3.1. Fabrication of Devices and Pyrolysis-Related Shrinkage of the GC Microelectrodes

**Figure 3:**
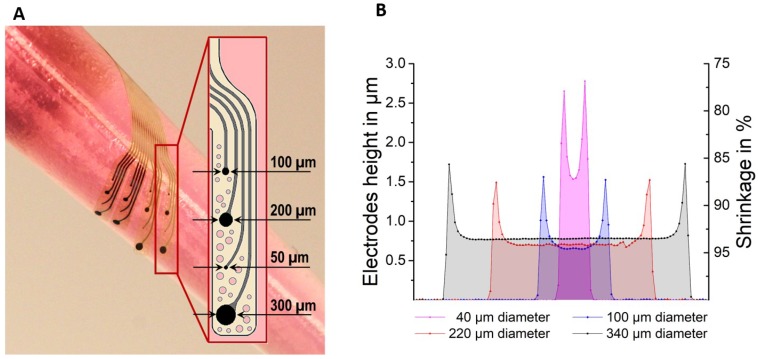
(**A**) Picture of an ECoG array after fabrication wrapped around a pipet with a diameter of about 8 mm. The inset shows one of the four PI ‘breathable’ or holed fingers with the different-diameter GC electrodes. (**B**) Profilometer data showing shape, height of GC electrodes (primary Y-axis) and vertical shrinkage in percentage (secondary Y-axis) of 40, 100, 220 and 340 µm diameter SU-8 structures after pyrolysis (1000 °C, in N_2_). The height of all the structures before pyrolysis was 13 µm. N = 5 samples per diameter.

The ultra-flexible ECoG devices with GC electrodes were successfully manufactured following the microfabrication steps listed in Figure 2. To ensure a good interlock of the materials (i.e., GC, PI and Pt), DLC and SiC were used as adhesion promoters [15,16] and—to achieve conformability of the devices over the curvilinear rat brains—the total thickness of the PI was set at 8 µm [29] and the electrode area was designed in a finger-like manner. Each finger was additionally designed to incorporate holes—and thus to be ‘breathable’—to allow the diffusion of biological fluids through the devices and improve the tissue-electrode contact. The devices were, in fact, able to naturally wrap around curvilinear bodies due to elasto-capillarity (Figure 3A, video in Supplementary Materials).

**Figure 4:**
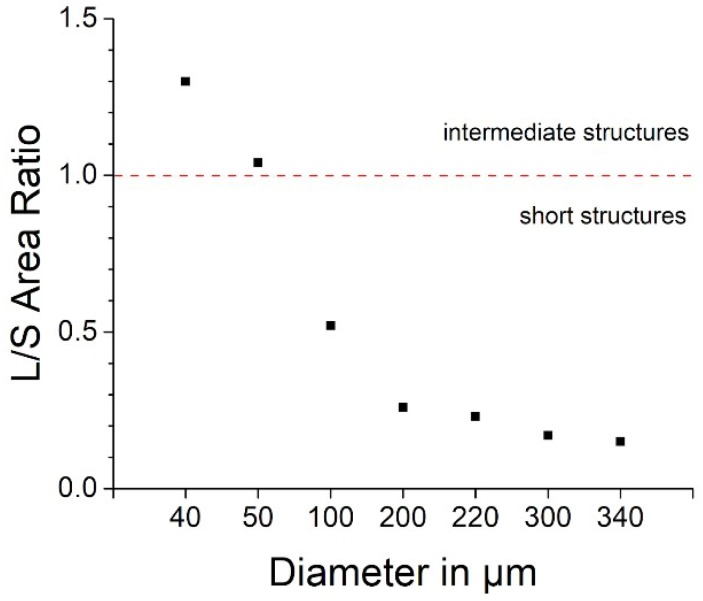
Lateral/surface area ratio calculated for not-pyrolyzed SU-8 cylindrical test structures with different diameters (ranging from 40 to 340 µm) and with a height of 13 µm before pyrolysis.

In addition to the ECoG electrodes, more disk-shaped test structures were made—on a separate silicon wafer using the same photoresist (SU-8) and the same protocol—for measuring their height before and after pyrolysis and quantifying pyrolysis-related shrinkage (Figure 3B). Similar to the ECoG electrodes, such test structures measured 13 µm in height before pyrolysis and had diameters of 40, 100, 220 and 340 µm. Results were obtained using a mechanical profilometer (Table S1, Supplementary Materials, where also the ratio between their lateral and surface area was calculated) and plotted in Figure 4, together with the values obtained from the actual ECoG electrodes. When this ratio is smaller than 1, the structures can be considered ‘short’ or low-aspect ratio structures; when it is greater than 10, instead, the structures are considered ‘tall’ or high-aspect ratio structures. When the lateral/surface area ratio is greater than 1 but smaller than 10, the studied geometries are known as ‘intermediate’ [20]. For low-aspect ratio geometries, vertical shrinkage (change in height) is expected to be more prominent than for intermediate and tall geometries. All the test structures and electrodes with diameter greater than 50 µm used in this study (i.e., 100, 200, 220, 300 and 340 µm) have lateral/surface area ratio < 1 and can thus be considered ‘short’ structures. Their vertical shrinkage ranges between 90 and 95%. The disks with diameters of 40 and 50 µm, on the other hand, have ratios of 1.3 and 1.04 (‘intermediate’ range) and their height after pyrolysis decreased by about 85% with respect to their height before pyrolysis. All the structures deformed during pyrolysis by sagging in the middle.

### 3.2. Electrochemical Characterization of the Devices (EIS, CV)

**Figure 5:**
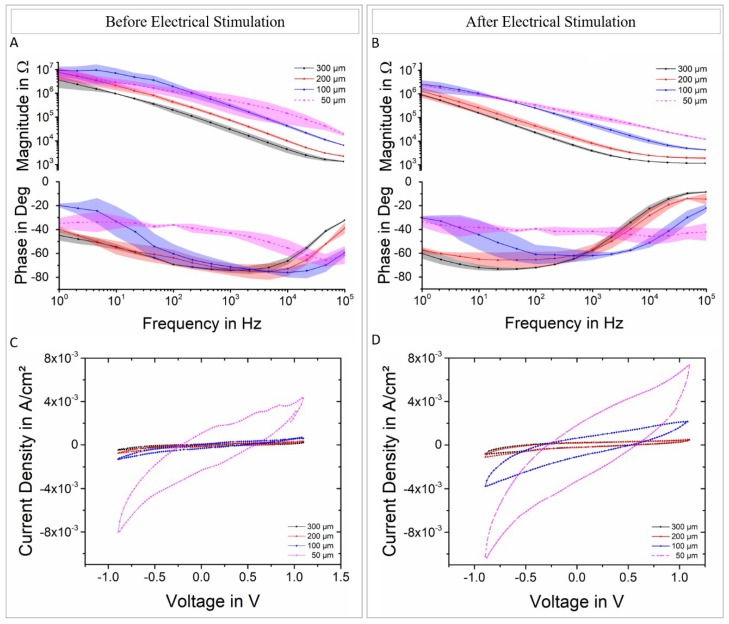
EIS (N = 4) and CV (representative cases) plots before (**A**,**C**) and after (**B**,**D**) electrical stimulation (10 million biphasic pulses maintaining a current density of about 0.15 mC/cm^2^). Shade regions represents standard deviation.

The different-diameter GC electrodes were electrochemically characterized before and after being electrically stimulated with 10 million biphasic pulses (Figure 5). During the stimulation, the phase period was set at 200 µs and the current density at 0.15 mC/cm^2^ throughout the array. Impedance spectra of the larger GC electrodes (200 and 300 µm in diameter) show a small reduction of the magnitude and a shift of the phase that becomes more resistive at high frequencies (10^4^ Hz and higher). Smaller electrodes (100 and 50 µm in diameter) exhibited a similar behavior but—in addition—the variability among those channels decreased after stimulation (i.e., standard deviation decreased, see Figure 5). All the electrodes were stimulated up to 10 million times to study the behavior of different geometries under stressing condition and to verify the achievement of the ‘steady-state’ previously seen in other studies [15,16]. Indeed, for all the electrode sizes, a decrease in impedance was observed (see Table S2 in Supplementary Materials for values at 1 kHz before and after stimulation) and in accordance with the referenced literature, although the 100 and 50 µm-diameter electrodes show unexpected high capacitance when compared to the larger ones.

It is well known that GC electrodes can be activated by electrochemical pretreatments (anodic, cathodic or both) and this phenomenon was, in fact, observed also in our previous work [15]. In particular, an increased surface roughness as well as an improved double layer capacity upon electrochemical and chemical activation was reported [30]. As expected, the main effect of the electrical stimulation protocol adopted in this study was to promote the electrochemical activation of all the GC microelectrodes. Thus, the higher capacitance observed for stimulated 100 and 50 µm-diameter electrodes can be explained in terms of the different operative conditions reached during the electrochemical pulses. In fact, as discussed in detail in the next section, the anodic voltage excursions decrease progressively from > +0.3 V, for 300- and 200-µm-diameter electrodes, to ~ +0.2 V or even lower for 100- and 50-µm-diameter ones. It is worth noting that the same charge density was applied during the stimulation but the less pronounced voltage polarization achieved by 100- and 50-µm-diameter electrodes was likely to promote a more effective electrochemical activation.

### 3.3. Electrical Stimulation and Voltage Transient

**Figure 6:**
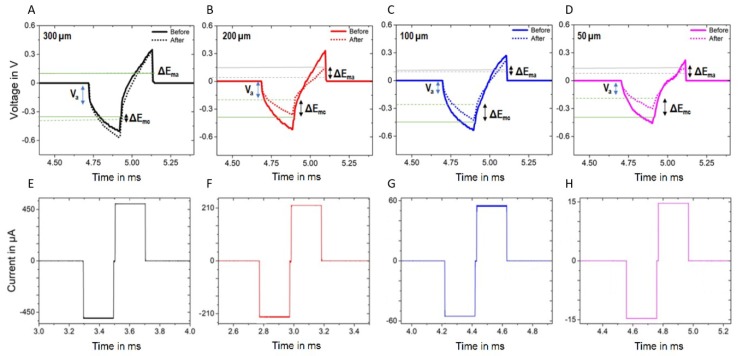
Voltage transient response of the four GC microelectrodes with diameters of 300 µm (**A**), 200 µm (**B**), 100 µm (**C**) and 50 µm (**D**), before and after stimulation (10 million pulses at 0.15 mC/cm^2^). (**E**–**H**) show the protocols used for the biphasic pulse stimulation of the different diameter GC electrodes.

Electrodes were stimulated with rectangular, charge-balanced biphasic pulses and the voltage transients were measured at the electrode’s interface before and after pulsing (Figure 6). For all the electrode sizes, the amplitude of the voltage transient stayed within the water window of GC (−0.9 to 1.3 V [14]) and the maximum negative voltage (E_mc_) reached before stimulation was similar for all the electrode sizes and ranged between the 0.54 V of the largest electrodes and the 0.46 V of the smallest ones. Such values decreased after stimulation for the 200-, 100- and 50-µm-diameter electrodes but not for the largest (300 µm). The difference between maximum negative potential before and after stimulation is shown in Figure 6 as ΔE_mc_, while the difference between the maximum positive (anodic) potentials is referred to as ΔE_ma_. Different-diameter carbon microelectrodes, with the exception of the largest (300 µm) ones, seemed to undergo similar change in negative potential (ΔE_mc_). The near-instantaneous voltage change (V_a_) associated with the Ohmic electrolyte resistance [31] decreased with the electrode size, though, and did not change significantly after stimulation. Surprisingly, electrical stimulation resulted in an improved charge injection limit for both 100 µm and 50 µm-diameter electrodes, as confirmed by their lower tendency to polarize after the initial ohmic voltage drop. What appears to be clear is that 300-µm-diameter electrodes did not exhibit any substantial change upon stimulation, at least in terms of voltage transients.

### 3.4. X-Ray Photoelectron Spectroscopy (XPS)

**Figure 7:**
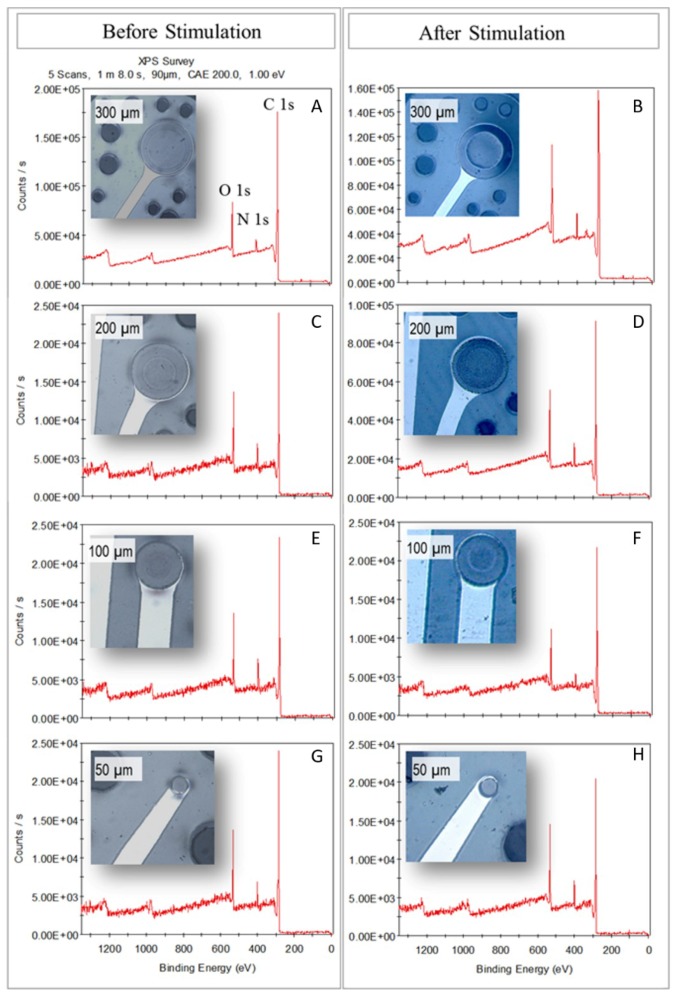
X-ray photoelectron spectroscopy (XPS) survey spectra of the four-diameter GC electrodes before (**A**,**C**,**E**,**G**) and after (**B**,**D**,**F**,**H**) stimulation. Insets show pictures of the electrodes with the correspondent diameter.

Elemental analysis of the four-diameter GC electrodes before and after stimulation was performed through XPS and survey spectra were recorded (Figure 7). The spectra revealed a C 1s signal of high intensity in addition to other two relatively high intensity peaks: N 1s and O 1s, residues of the pyrolysis process. Although the spectra of all the electrodes looked very similar and demonstrated that no other species other than carbon, nitrogen and oxygen were present on the electrodes surface, by looking at the percentages associated with the C 1s, O 1s and N 1s peaks (table in Supplementary Materials), it is noticeable that the pristine electrodes can be divided into two groups: small (50 and 100 µm in diameter) and large (200 and 300 µm), where the two groups mainly differ in percentage of carbon compounds and oxygen content. Such group division is no longer detectible after electrical stimulation. However, it must be noted that, due to the destructive nature of the XPS test, the ‘stimulated electrodes’ used for the analysis could not be the same ones used for the ‘pristine electrodes’ analysis. This also explains the random presence of Si residues (due to the fabrication method itself) on some carbon microelectrodes. Details of the relative quantities (in %) of the different compounds found during the analysis with associated eV values and possible assignments are listed in Table S3.

### 3.5. In Vivo Experiments

**Figure 8:**
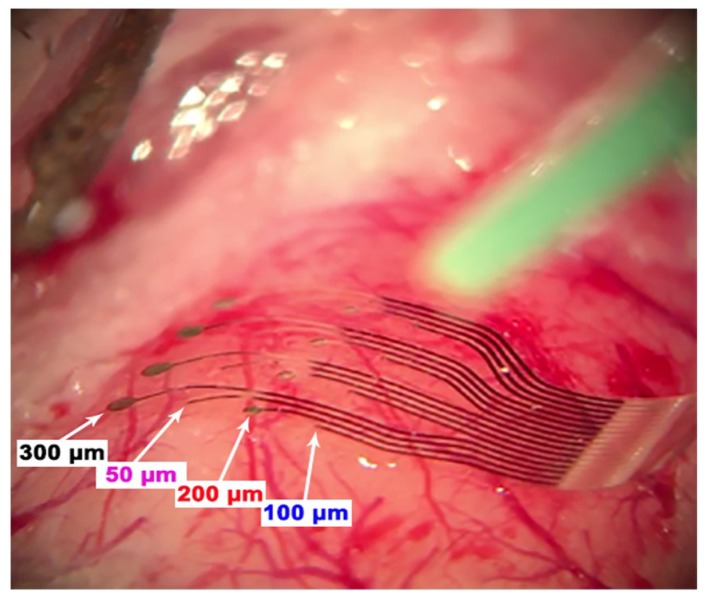
ECoG device implanted over the left barrel cortex of a rat during an acute experiment. The array perfectly adapts to the curvature of the cortex enabling strong biotic/abiotic interface contact.

The conformable ECoG arrays were placed over the rat barrel cortex of five rats in several positions (an example in Figure 8) to test the ability of each electrode geometry to record neural activity independently of its distance from the signal source. The devices were tested first by simultaneously stimulating all the whiskers and then only single whiskers, with the aim of studying also the electrodes’ capability to detect and discriminate the response evoked by one single barrel of the rat cortex [32,33].

**Figure 9:**
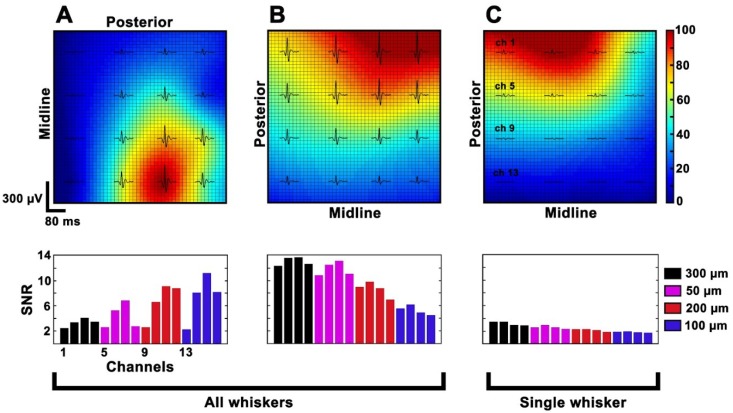
Representative interpolated maps of the averaged SEPs, first row recorded in different positions (A-B) or in the same position but stimulating all and single whiskers (B-C). The colormap reflects the SEP sizes normalized in the range [0, 100] independently for each experimental condition. The second row reports the corresponding SNR histograms, which show how the same electrode can take high or low values depending on its position or intensity of the signal.

For each position, the location of the largest recorded SEP, identified as the signal source, moves on the electrode color-coded map consistently with the repositioning of the array on the cortex (Figure 9). When placed close to the signal source, in fact, each electrode diameter is able to record the greatest peak-to-peak amplitude. Consequently, the resulting signal-to-noise ratio (SNR) values (Figure 9, bottom row) do not depend on the electrodes’ dimensions but are highly correlated to the signal source location and to the intensity of the signal. On the other hand, when only single whiskers are stimulated (Figure 9C), smaller SEP amplitudes and lower SNR values are expected due to the reduced population of neurons involved in the task (with respect to the all-whisker’s case of Figure 9A,B) rather than to the electrodes themselves.

**Figure 10:**
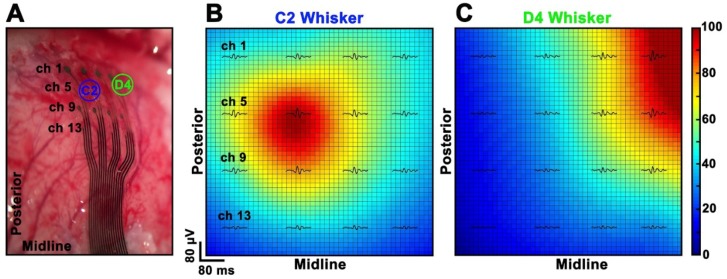
(**A**) Picture of the array over the barrel cortex where the position of the barrels of interest are overlaid. (**B**,**C**) Interpolated maps of the averaged SEPs obtained after stimulating the C2 and the D4 whiskers showing the expected somatotopic shift. The colormap reflects the SEPs sizes normalized in the range [0, 100] independently for each experimental condition.

Moreover, when stimulating one single whisker, the expected somatotopic shift of the neural response, which follows the barrel cortex organization, was defined in concordance with the selected whisker and discriminated by every electrode diameter when on top of the barrel of interest (Figure 10). 

**Figure 11:**
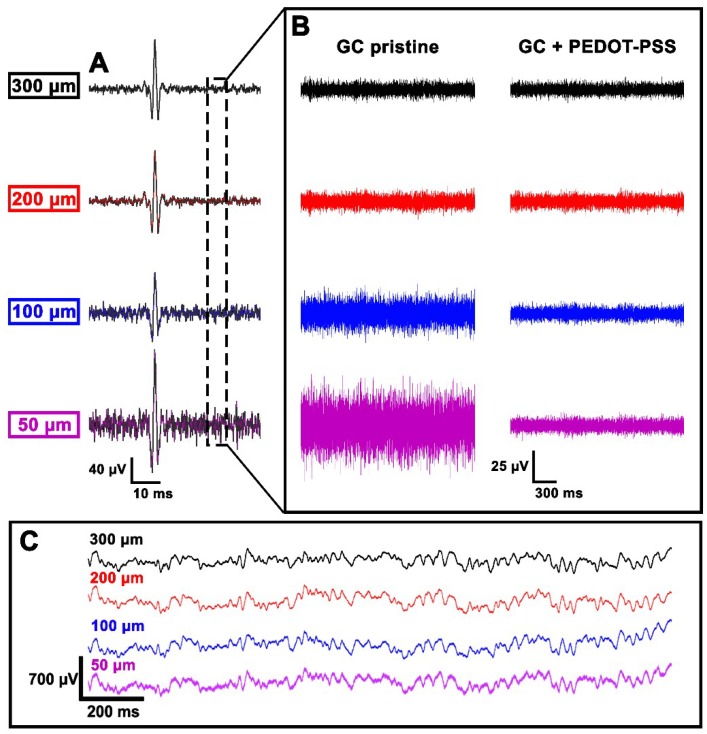
Representative continuous traces of one trial (**A**) recorded by all the electrode diameters after high-pass filtering the data above 200 Hz. The grey box (**B**) represents the magnification of a small time window of spontaneous activity in order to highlight the background noise for every electrode diameter before and after PEDOT:PSS deposition. Box (**C**) shows ECoG raw data recorded from pristine GC electrodes of various diameter.

When dealing with SEP recordings, no relevant difference between the recording performances could be associated to the different electrode diameters. Differently, a meaningful distinction between the diameters was noticed in the high frequency domain (Figure 11). In concordance with the trend emerged from the electrochemical characterization, the high frequency background noise band increased with the decrease of the electrode diameters, except for the transition between 300 and 200 µm, which did not show significant changes. In order to address the noise band increase, electrochemical deposition of PEDOT:PSS onto pristine GC electrodes was performed. As shown in Figure 11, a significant background noise reduction was observed for the 50- and 100-µm-diameter electrodes after the coating, making them comparable to the larger electrodes (200 and 300 µm in diameter) that remained basically identical (for amplitude ranges see Table S4 of the Supplementary Materials). The correlation between the SNR value associated with one electrode and its relative position to the signal source was observed in all five rats.

## 4. Discussion

This study demonstrates that miniaturization of GC ECoG electrodes and conformability of such implants can be reached by tuning the implant design. A more conformable device allows the achievement of optimum structural biocompatibility without the need of a two-axis soft material. When conformability is achieved, in fact, the tissue-electrode interface improves and allows better implant performance, independently from the electrode diameters (in our 50 to 300 µm range). As expected, short structures (100, 200 and 300 µm in diameter) during pyrolysis shrink differently from the intermediate structures (50 µm). The fact that we pyrolyzed them in N_2_ explains the massive vertical shrinkage, and the fact that the electrodes are not freely standing—but attached to a substrate—surely explains the limited shrinkage in diameter. The amount of impurities, on the other hand, is higher in the intermediate electrodes and they seem to undergo higher activation (and increase in capacitance most likely due to removal of residues from the surface). Electrodes with 100 µm diameters behave in some cases like those with 50 µm diameters, and in others like the larger electrodes, probably due to the fact that they are at the ‘edge’ between the two categories (i.e., short vs intermediate). Looking at the electrochemical characterization of the electrodes and at the XPS measurements before and after electrical stimulation, it can be stated that the implants stayed intact and no delamination occurred, as no metal is present in the spectra and the CVs remained featureless. Additionally, the larger structures (200 and 300 µm in diameter) are the only ones to have some kind of graphitic component and can be considered the ‘purest’ ones: the degassing of non-carbon elements is easier in short structures thanks to their smaller volume-to-area ratio. Such structures are also more similar to GC pyrolyzed at higher temperatures (>1000 °C) and thus electrochemically more inert. In order for the smaller electrodes to be ‘purer’ (more similar to the larger electrodes), they should be made thinner and thus be categorized as ‘short’ structures.

The in vivo performance evaluation was conducted with the aim of understanding how electrode diameter can affect the neural signals recorded during acute experiments. In our study, larger neural responses seem mainly related to the distance of the electrodes from the signal source rather than to the electrode diameter (see Figure 9). Indeed, a large deviation in SNR values of identical electrode diameters is reported (Figure 9A). This effect is clearly related to the position of the array over the barrel cortex: very small SNR values (channels 1, 2, 3, 4, 5, 8, 9, 13) correspond, in fact, to the electrodes distant from the center of the barrel cortex (the most posterior or medial ones). Further evidence is shown in Figure 9B, where the results obtained after a 90° rotation of the array are reported. Here, it can be seen that the best-performing electrodes are again the closest to the signal source, independently from their diameter. All the tested diameters have eventually proved to be capable of reliably mapping the distribution of cortical sensory processes evoked by peripheral stimulation (see Figure 10). However, in terms of high frequency background noise (see Figure 11), the 50 µm-diameter electrodes were demonstrated to be the worst-performing ones and to have larger noise bands than the other diameters. Since the SEPs represent a massive neural response (thousands of µV or even in the mV range), the larger noise band of the 50 µm electrodes (circa 30–60 µV) has no remarkable consequences for this type of recording. This is confirmed also by the resulting SNR values (Figure 9), which were also calculated in the high-frequency range. Although this difference is not critical when dealing with slow and big neural responses (i.e., SEPs), it may be a non-negligible issue when aiming to record fast and small neural activities (i.e., spikes) because they could remain totally embedded in the background noise and be indistinguishable. As well known in the literature (and also according to our past experience [14]), by coating the electrodes with PEDOT, it is possible to significantly reduce their background noise band. This allowed us to level the differences among the different-diameter electrodes and to obtain similar noise levels for small and large ones (see Figure 11 and Table S4 of the Supplementary Materials). This way, the detection of very small and fast signals may be possible [9,34]. The devices were perfectly able to adapt to the curvature of the cortex and to establish a strong contact with the brain tissue (see Figure 8) thanks to their flexibility, and finger-like and ‘breathable’ substrate. Due to these features, the SNR improved and the effort spent in positioning the array to obtain a good contact with the cortex is highly reduced (see Video S1 of the Supplementary Materials). This aspect of structural biocompatibility should not be underestimated with respect to reduction in foreign body reaction and chronic functionality.

## 5. Conclusion

Our study demonstrates that pyrolysis-dependent shrinkage has an effect on the in vitro and in vivo performance of GC electrodes. When the electrodes are intermediate or tall (in our case the 50-µm-diameter electrodes) before pyrolysis, they are expected to withhold some impurities because of the limited top surface area available for degassing during pyrolysis. They will therefore shrink less in height than the others. The presence of impurities seems to be correlated to the electrode’s charge storage capacity (CSC) increase after electrical stimulation: our 50-µm-diameter electrodes show, in fact, a larger increase in CSC with respect to the larger ones. This is probably due to the increase of surface roughness subsequent to electrical stimulation and residue elution. In vivo results show that, when used to record signals below 300 Hz, the electrode’s performance depends on its vicinity to the signal source rather than on its geometry and dimension. When used to record in the physiological high frequency band (above 200 Hz), instead, the smaller electrodes appear to be noisier than the larger, unless coated with PEDOT:PSS. The finger-like structure of the implants and their thin and conformable substrate are key factors for improving the structural biocompatibility of the ECoG arrays. These features, however, strictly depend on the target anatomical area, on the application and on the animal model under investigation, as well as on the implantation procedure. Careful selection of design parameters can lead to reduced gaps between the implant’s material and the host tissue, and eventually to good signal-to-noise ratios in chronic applications. 

## Supplementary Materials

The following are available online at http://www.mdpi.com/1996-1944/11/12/2486/s1, Table S1: Geometrical values for disk-shaped glassy carbon structures. All samples had a height of 13 µm before pyrolysis; Table S2: Values of impedance at 1 kHz and charge density before and after stimulation. N = 3; Table S3: Relative quantities (%) of different compounds for various diameter pyrolyzed carbon electrodes; Table S4: Observed amplitude range of the background noise for every electrode diameter before and after PEDOT:PSS deposition; Video S1: Glassy Carbon ECoG Electrodes on Ultra-Conformable and Finger-Like Polyimide Substrate on Rat Brain.
