# Supplementary material for: Glassy Carbon Electrocorticography Electrodes on Ultra-Thin and Finger-Like Polyimide Substrate: Performance Evaluation Based on Different Electrode Diameters
Source: Materials (Basel). 2018 Dec 7;11(12):2486. doi: 10.3390/ma11122486 (PMC6316905; doi:10.3390/ma11122486)
Supplement: Supplementary file 1 [file materials-11-02486-s001.zip › materials-397789-SI.docx]

**Supplementary Materials: Glassy Carbon Electrocorticography Electrodes on Ultra-Thin and Finger-Like Polyimide Substrate: Performance Evaluation Based on Different Electrode Diameters**

Maria Vomero ^1,2,^*^,†^, Elena Zucchini ^3,4,†^, Emanuela Delfino ^3,4^, Calogero Gueli ^1^, Norma Carolina Mondragon ^1^, Stefano Carli ^3^, Luciano Fadiga ^3,4^ and Thomas Stieglitz ^1,2,5^

**Table S1.** Geometrical values for disk-shaped glassy carbon structures. All samples had a height of 13 µm before pyrolysis.

| **Diameter**  **in µm** | **Surface Area**  **in µm^2^** | **Lateral Area in µm^2^** | **Lateral/Surface Area Ratio** | **Average Height After Pyrolysis in µm** |
| --- | --- | --- | --- | --- |
| 40 | 1256.64 | 1633.63 | 1.30 | 1.75 |
| 50 | 1963.5 | 2042.04 | 1.04 | N/A |
| 100 | 7853.98 | 4084.07 | 0.52 | 1.00 |
| 200 | 31415.93 | 8168.14 | 0.26 | N/A |
| 220 | 38013.27 | 8984.95 | 0.24 | 0.90 |
| 300 | 70685.83 | 12252.21 | 0.17 | N/A |
| 340 | 90792.03 | 13885.84 | 0.15 | 1.10 |

**Table S2.** Values of impedance at 1 kHz and charge density before and after stimulation. N = 3.

| **Diameter in µm** | **Impedance @ 1 kHz**  **before Stimulation in kΩ** | **Impedance @ 1 kHz**  **after Stimulation**  **in kΩ** | **Charge Density**  **before Stimulation**  **in C/cm^2^** | **Charge Density**  **after**  **Stimulation**  **in C/cm^2^** |
| --- | --- | --- | --- | --- |
| 50 | 514.8 ± 376.4 | 115.5 ± 28.9 | 0.107 ± 0.079 | 0.302 ± 0.025 |
| 100 | 305.7 ± 78.5 | 50.7 ± 11.8 | 0.029 ± 0.008 | 0.088 ± 0.016 |
| 200 | 75.5 ± 9.4 | 8.3 ± 1.5 | 0.011 ± 0.005 | 0.024 ± 0.005 |
| 300 | 31.1 ± 9.3 | 3.9 ± 0.5 | 0.007 ± 0.002 | 0.017 ± 0.004 |

**Table S3.** Relative quantities (%) of different compounds for various diameter pyrolyzed carbon electrodes.

| **Atomic %** | | | | | | | | | |
| --- | --- | --- | --- | --- | --- | --- | --- | --- | --- |
| **Electrode Diameter**  **in µm** | | **C 1s** | **C 1s** | **C 1s** | **C 1s** | **O 1s** | **O 1s** | **N 1s** | **Si 2p** |
| Pristine | 300 | 26 | 40.3 | 9.6 | 5.6 | 10.2 | 2.7 | 3.6 | 1.4 |
|  | 200 | 19.7 | 48.6 | 8.9 | 5.5 | 9.5 | 2.7 | 2.8 | 1.4 |
|  | 100 | 0 | 68.6 | 0 | 10 | 12.6 | 1.6 | 7.3 | 0 |
|  | 50 | 0 | 67.2 | 1.4 | 10 | 12.7 | 1.4 | 7.3 | 0 |
| Stimulated | 300 | 0 | 54.7 | 12.3 | 9 | 11 | 6 | 5.6 | 1.5 |
|  | 200 | 0 | 63.7 | 5.4 | 8.9 | 12.6 | 2.7 | 5.7 | 1.1 |
|  | 100 | 0 | 67.3 | 6.3 | 7.3 | 10.4 | 3.2 | 4.2 | 1.3 |
|  | 50 | 0 | 59.9 | 5.8 | 9.4 | 13.5 | 2.7 | 7.2 | 1.5 |
| eV |  | 284.4 | 285 | 286 | 288.5 | 532.2 | 533.6 | 400 | 102 |
| possible assignments |  | graphite | C–H | C–O/  C–N | C=O | C=O/  SiO_x_ | C–O | C–N | SiO_x_ |

**Table S4.** Observed amplitude range of the background noise for every electrode diameter before and after PEDOT:PSS deposition.

| **Electrode Diameter in µm** | **GC Pristine in µV** | **GC + PEDOT:PSS in µV** |
| --- | --- | --- |
| 300 | 10–20 | 5–15 |
| 200 | 10–20 | 5–15 |
| 100 | 15–30 | 5–15 |
| 50 | 30–60 | 5–15 |

**Video S1.** Glassy Carbon ECoG Electrodes on Ultra-Conformable and Finger-Like Polyimide Substrate on Rat Brain.
